# Development and validation of an instrument to measure and manage organizational process variety

**DOI:** 10.1371/journal.pone.0206198

**Published:** 2018-10-23

**Authors:** Sarah Zelt, Jan Recker, Theresa Schmiedel, Jan vom Brocke

**Affiliations:** 1 Institute of Information Systems, University of Liechtenstein, Vaduz, Principality of Liechtenstein; 2 Faculty of Management, Economics and Social Sciences, The University of Cologne, Cologne, Germany; 3 QUT Business School, Queensland University of Technology, Brisbane, Australia; 4 Institute of Information Systems, University of Applied Sciences Northwestern Switzerland, Switzerland; University of Copenhagen, DENMARK

## Abstract

Organizational processes vary. Practitioners have developed simple frameworks to differentiate them. Surprisingly, the academic literature on process management does not–it typically strives for one method to manage all processes. We draw on organizational information-processing theory to systematically develop a new, theoretically motivated classification model for organizational processes. We validate this model using survey data from 141 process practitioners of a global corporation. We derive three distinct types of processes and demonstrate that an understanding of process variety based on process dimensions can differentiate processes better than existing frameworks used in practice. Our findings can enable process managers to make informed decisions and serve as a basis for contingent process management.

## Introduction

Organizational processes, “repetitive patterns of interdependent action carried out by multiple actors" [1], can vary substantially. In manufacturing, for example, processes differ alongside physical properties (such as machinery), dimensional tolerance (size and form) or outcomes (e.g., defect rates). Outside of manufacturing, many different types and variations of processes are also documented. This is quite intuitive: The process of drug prescription in healthcare is clearly different from surgery. Procurement is different in many ways from order fulfillment.

To absorb these differences, practitioners use simple frameworks to classify their processes. Text- and management handbooks for process management speak of primary versus secondary activities [2] or core, management, and support processes [3]. These frameworks are widely adopted in practice.

In the academic literature, however, the differences that make up “process variety” have not yet been conceptualized in a consistent or systematic way. For example, processes with “higher complexity” are said to be harder to standardize [4], processes which are “interdependent” require more detailed planning [5], and processes with low “variability” can be supported more cost-efficiently with enterprise systems [6]. Moreover, where such variety-related concepts have been used, they have been put in relation to only selected and isolated process management decisions, such as virtualization, systems implementation or standardization [4, 6, 7].

By contrast, many scholars contend that business process management often strives for one best method [8; 9], in the tradition of scientific management [10]. However, in order to make informed decisions and to prevent wasted efforts, process management research needs to understand and conceptualize process differences and classify processes into meaningful categories [11], which we intend to do in this paper.

We review the literature on process differences and develop a new process classification model grounded in organizational information-processing theory (OIPT) [12]. We develop a rigorous measurement instrument that allows describing and classifying business processes in practice. We then demonstrate how our model allows differentiating processes and how it supports the derivation of appropriate management methods better than existing frameworks.

We proceed as follows: we first review academic and practitioner literature on process dimensions and frameworks. We then develop a new classification model and measurement instrument on basis of OIPT. We report on our application of this instrument within one case organization to evaluate our classification model by comparing it to existing process frameworks. The paper ends with a discussion, implications for research and practices, as well as limitations and review of contributions.

## Background

### Process variety in theory

Organizations feature and enact many different processes. The process of handling a customer order is different in many ways from the process of granting employee leave. Intuitively, these two processes differ at least in terms of value creation potential, complexity, rigidity, variability and potentially other dimensions.

Yet, in the academic literature, these and other dimensions of process variety have only partially and selectively been examined and/or linked to process management decisions. For example, Schaefermeyer et al. [4] distinguished processes based on their degree of *complexity* (operationalized through the degree of non-routineness, variety, and uncertainty) and found that process complexity negatively influences process standardization. Mani, Barua, and Whinston [13, 14] proposed that process complexity (operationalized as processes with low levels of analyzability and high levels of variety) influences business process outsourcing success. While both contributions examine process complexity, there seems to be no common understanding of how complexity is conceptualized or operationalized–research teams used different process dimensions when referring to complexity [5, 15, 16].

A similar confounding of conceptualizations can be found when examining the often-used term of *knowledge-intensity* of processes [17, 18]–another dimension alongside which processes may vary. Knowledge-intensive processes contain the transfer of knowledge between process participants and purportedly require different management methods due to their unpredictable tasks [17, 18]. Davenport [19] distinguishes knowledge-intensive processes based on the *degree of expertise* and *level of coordination* that is required to execute the process. Yet, other researchers suggest that knowledge-intensive processes are “artistic” [20] or “creative” [21] as opposed to *machine-intensive* or *(partly) automated* [22, 23].

These examples illustrate that the process management literature acknowledges the existence of process variety, yet to date no consistent conceptualization or operationalization exists. Moreover, business process management as a method still retains the basic premise that all processes can be managed with the same set of methods and procedures [8, 9]. Organizations are often aware of this challenge [24], which is why process variety has been acknowledged and partially accounted for through a variety of process frameworks popular in management practice.

### Process variety in practice

Practitioners have for a long time understood that not all processes matter in the same way. The literature practitioners engage with features a variety of frameworks (for an overview see [25]) to differentiate, for example, manufacturing or service processes. For example, the product-process matrix [26, 27] suggest four options on how to structure manufacturing processes (job shop, batch, assembly line, and continuous flow) which differ in terms of their labor-intensiveness, flexibility, and efficiency. Other widely adopted process frameworks include the value chain model by Porter [2], which differentiates between primary and secondary activities: primary activities directly contribute to an organizations value creation while secondary activities support primary activities in being more efficient and effective. Examples for primary activities are inbound and outbound logistics, operations, marketing, sales, and services while secondary activities are procurement, human resource management, infrastructure, and technological development. Similarly, Ould [3] differentiates between core, management, and support processes. While core processes and support processes are similar to primary and secondary activities, management processes are strategic processes focusing on goals, monitoring, and control.

Building on this high-level differentiation of processes into core, management, and support processes, practitioners have developed further, rather functional process frameworks such as the *American Productivity and Quality Center (APQC)* [28] or the ISO 9000 typology of organizational processes [29].

In the ISO 9000 quality management standards, processes are distinguished into management, realization, resource management, and measurement, analysis and improvement [29]. The framework of APQC is one of the most frequently applied process frameworks and distinguishes between five core processes (e.g., develop and manage, market and sell, and deliver products and services) and seven management and support processes (e.g., manage human capital, information technology, and financial resources). Both frameworks are closely related to traditional organizational functions and do not consider similarities or differences of processes within or across functions.

These practical frameworks have in common that they reduce the complexity of process management by representing the plethora of organizational processes through a manageable and limited number of categories, which in many cases distinguish processes based on their value creation potential. In addition, these frameworks allow comparing organizational processes, so they lay the foundation for internal benchmarking.

Our conclusion from the review of the academic and practitioner literature on process variety is threefold:

1. The academic literature is inconsistent and isolated in how it treats process variety: while singular dimensions associated with variety have received attention, there is no general, coherent framework and measurement instrument.
2. Practitioner frameworks have some apparent usefulness, yet it is difficult to support decision-making in process management and to derive distinct management recommendations from these because they are too abstract: within each category (e.g. core processes), a large variety of different processes exists;
3. All of the existent frameworks are descriptive but neither explanatory nor prescriptive: the frameworks cannot answer the question how (i.e., on which dimensions) processes differ; nor do they suggest methods for how to deal with different processes.

### Organizational information-processing theory

When trying to understand process variety and its management implications, we thought it valuable to look into organizational design research, in which differences between whole organizations have been examined and comprehensive theories developed (e.g., see [30, 31]). One such example is organizational information-processing theory (OIPT) [12], a theory which has been successfully applied by multiple researchers as a foundation to conceptualize process differences [14, 32, 33]. Thus, OIPT became our starting point.

OIPT [12] views organizations as information-processing systems. The theory states that the characteristics of an organization’s basic task lead to varying information-processing requirements and that distinct management approaches are required to deal with these requirements. Researchers who applied OIPT to their study have analyzed which task characteristics influence information-processing requirements, most frequently suggesting task characteristics such as task variability, analyzability, interdependence, and differentiation (e.g., see [34, 35, 36]). These characteristics are thought to determine the amount of information that employees need to process during task execution.

The theory also explains why different tasks require a different management approach: In order to increase the effectiveness and efficiency of an organization, information-processing requirements must fit to information-processing capacity available within an organization. The information-processing capacity, on the other hand side, is influenced by organizational structures or management practices. Thus, OIPT suggests that organizations have to implement structures or management practices to deal with the information-processing requirements that are caused by task characteristics.

OIPT has not only been applied on an organizational level but has also been transferred to organizational subunits and processes [14, 32, 33]. Processes consist of many interrelated tasks and can be viewed as information-processing systems, so OIPT has been used to explain why process dimensions influence the success of process management practices [14, 33]. A systematic literature review of process dimensions (self-citation omitted for review) has shown that the many different dimensions found in the academic literature can indeed be grouped and structured according to OIPT and its proposed dimensions. Thus, OIPT helps to understand process differences and it can be used as a theoretical basis for developing a new process classification model. What remains open, however, is how to operationalize process differences and how to develop an instrument that can measure process differences within one organization.

### Research objective

While processes in organizations are quite diverse, both the frameworks developed in practice and the varying conceptualizations of process differences discussed in academic literature insufficiently encompass and measure this variety. However, to make informed decisions and to prevent wasted efforts, it is important to both understand and measure process differences [11, 37]. Thus, the research objective of this paper is to identify and operationalize critical process dimensions in a new classification model. Thus, we intend to develop a reliable and valid instrument that measures process variety. We hereby draw on OIPT on the basis of the assumption that processes (as organizational meso-level systems) can be viewed as information-processing systems [14].

## Method

**Fig 1:**
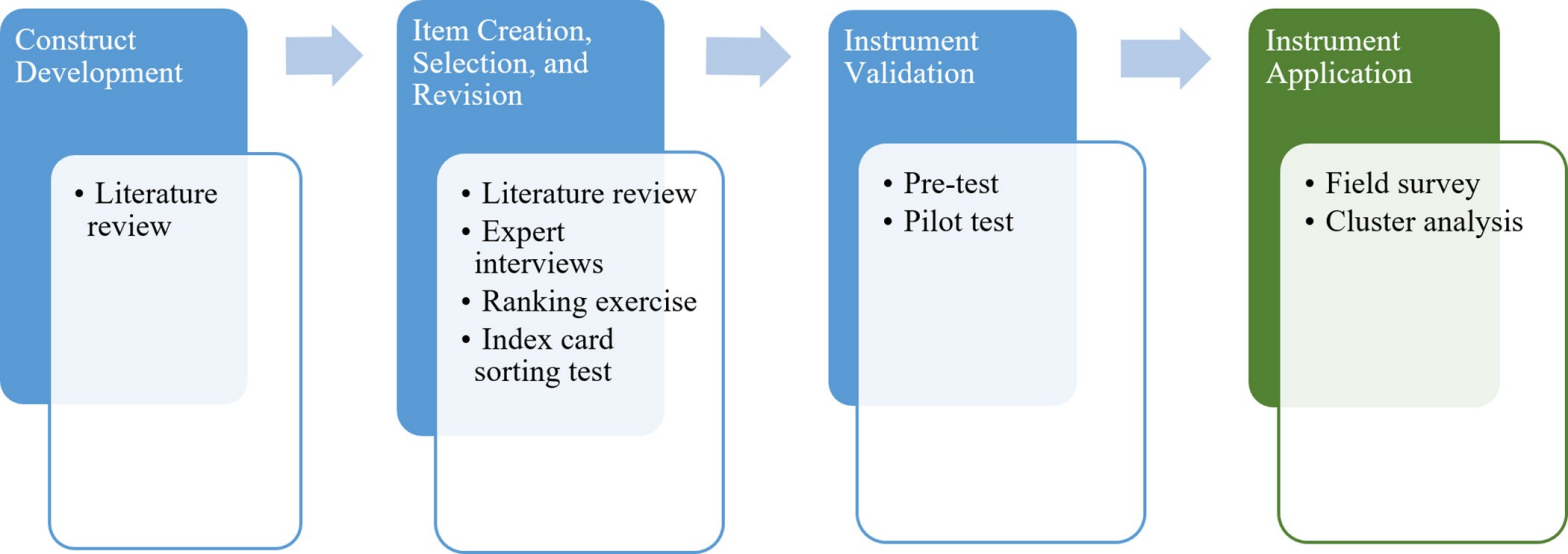
Research approach.

To develop an instrument that measures process variety and its constituent dimensions as a basis for a new process classification model, we followed a multi-stage approach building on well-recognized approaches for instrument development and validation [38, 39, 40, 41]. Fig 1 visualizes the methods we employed to identify and operationalize process dimension constructs and to ensure reliability and validity of our instrument.

First, we derived critical process dimensions from existing research building on information-processing theory (construct deveopment). We then conducted a literature review as well as expert interviews to identify a pool of candidate items that can be used to measure our constructs (item creation). We then asked experts to evaluate the extent to which each item measures the intended construct which allowed ranking the items and selecting most suitable ones (item selection). In a card sorting exercise, experts then sorted the items into categories helping us to further improve the items and to get a first indication of their convergent and discriminant validity (item revision). After refining the items, we prepared a first version of the survey that we pre-tested with experts to further improve the understanding and clarity of the overall survey instrument (instrument validation). We also collected data in a pilot test and performed statistical analysis assessing reliability and validity of our instrument. Last, we conducted a field survey (instrument application) in which we measured process variety in a global process-centric organization. As a post-hoc analysis, we performed a cluster analysis to identify frequently occurring types of processes showing a similar pattern of process characteristics. The research project and instrument were approved by the Rectorate of the University of Liechtenstein (https://www.uni.li/en/university/about/organization). Data collection procedure and instrument were also approved by the Global Data Privacy and Protection Office as well as the BR-ITP committee of the Workers Council of the case organization. The results of each stage are explained in detail in the next section. Measurement development.

### Construct development

The first step was to identify critical process dimensions that can be used to describe process differences. Our starting point is the view that business processes consisting of many connected tasks can be understood as information-processing systems [13, 14, 33]. To substantiate the validity of this view, we carried out a structured literature review on process differences and mapped these to the OIPT dimensions (self-citation omitted for review). Our mapping showed that all reported process characteristics can be mapped to five process dimensions: the degree of variability, interdependence, knowledge-intensity, differentiation, and importance. We define each as follows.

**Variability** describes the degree to which process inputs, steps, or outputs during execution are variable and difficult to predict in advance [12, 34]. Thus, process execution can vary a lot from one instance of a process to another. As an example, administration or craft work usually show low levels of variability, while research, strategic planning, or engineering are highly variable [30]. This dimension has been shown to be closely related to other dimensions used in process management literature (self-citation omitted for review) such as the degree of input variety [42], output variety [43], repeatability [17], routinization [16], and predictability [17].

**Interdependence** describes the degree to which people or departments involved in process execution depend on others in accomplishing their work [35, 44]. Interdependence is one of the most frequently mentioned process dimensions in process management literature [5, 19] and distinguishes processes in call centers with low levels of interdependence and processes such as software development or research, which often involve many process participants depending on each other in accomplishing their work. Other process management researchers referred to it as the degree of collaboration [19, 45], iterativeness [19], or linearity [15].

**Knowledge-intensity** describes the degree to which personal judgment as opposed to computational procedures is required during process execution [34, 36]. While administration, work on an assembly line, and engineering can follow computational procedures for which mostly one right answer can be derived, there are processes such as craft work, research, or strategic planning for which knowledge or personal judgment is quite important [30]. This dimension reflects the frequent differentiation into knowledge-intensive and non-knowledge-intensive business processes [17]. In OIPT literature, this dimension has also been called “analyzability” [34, 36] but as the term knowledge-intensity is more frequently used in process management literature [17, 18], we refer to this dimension as knowledge-intensity instead of analyzability.

**Differentiation** describes the degree to which people with different backgrounds, experience, goals, and priorities are involved in executing a process [34, 36]. Some processes involve experts from only one or very similar areas (e.g., financial reporting, engineering), while others show much differentiation between the involved departments or roles (e.g., research, strategic planning). In process management literature, authors described process differences in terms of process boundaries (process within vs. between organizations) [46, 47], number of process participants [43], and degree to which also customers are involved in the process [43, 48]. These dimensions describe to what extent different people are involved in a process, which we summarized under the process dimension differentiation.

Last, **importance** has been identified as a critical process dimension both in practitioner process frameworks [2, 3] and in research that applied OIPT to the field of process management [13, 33]. While the other four process dimensions rather refer to how the process is being executed, process importance describes the extent to which a process itself is valuable to the firm and impacting its competitiveness [13]. The frequent differentiation between core, management, and support processes in practitioners’ frameworks [3] shows that processes differ in their degree of value contribution or importance. Process importance also seems to influence information-processing requirements and thus, impact business process outsourcing [13] and process visibility [33].

Based on OIPT and research that applied OIPT to process management, we consolidate process differences through the dimensions process variability, interdependence, knowledge-intensity, differentiation, and importance. These dimensions served as the constructs on which our process classification model is based. Thus, we operationalized these constructs in a second step.

### Item creation, selection, and revision

#### Item creation

In instrument development, indicators used to measure a construct need to show content validity which can be defined as the degree of correspondence between the items selected to constitute a summated scale and its conceptual definition [49]. Thus, a conceptual definition of each construct of interest is required and a list of candidate items that represent the dimensions of the construct [39]. Based on the conceptual definitions of our five process dimensions, we conducted a literature review to identify suitable candidate items. We searched for sources that applied organizational information-processing theory to different contexts and for sources that operationalized one or multiple constructs that we intended to measure.

Most of the existing items were developed for other contexts (organizational tasks, subunit tasks, or projects) and had to be adapted to the process context. As an example, Whitey, Daft, and Cooper [50] operationalized task variability and task analyzability. The authors hereby combined scales originally developed by Daft and Macintosh [51] and Van de Ven and Delbecq [52] which we also included into our item pool. Chang and Chiu [53] analyzed the nature of projects by examining five sources of uncertainty and equivocality. We re-used their interdependence items as well as their differentiation items, as the construct definition showed similarities to our construct definitions (besides the unit of analysis).

For each construct, we adapted the original items so that they fit our conceptual definition and unit of analysis (i.e., processes). As an example, we changed the original item “During a normal month, how frequently are exceptions expected to arise from doing the project” [53] to “There are frequent exceptions expected to arise during process execution” or “To what extent is there a clearly defined body of knowledge of subject matter which can guide you in doing your work” [50] to “There is a clearly defined body of knowledge which can guide process participants”.

To ensure that we speak the language of process management practitioners, we conducted interviews with seven process management experts from the target organization in which we intended to apply the instrument, in which we asked them to describe characteristics of processes. We contacted the experts via a central team which is responsible for process management. These interviews served as an intermediate step and helped us in adapting the existing items to the process context in the target organization. In addition, we developed new items by using phrases or sentences that the practitioners used in describing processes such as “a lot of colleagues or other workspaces are involved in this process” (for differentiation), “I am depending here on other teams” (for interdependence), or “processes which are more static—you have to follow a clear guidance” (for analyzability). In adapting and creating the items, we followed the recommendation of Ajzen and Fishbein [54] to include the actual behavior (e.g., “frequent information exchanges”), a target (e.g., “between process participants”), a context (e.g., “for the/during process execution”), and where applicable also a time (e.g., “from one time to the other”). In addition, all items were formulated in simple ways, precisely avoiding “and” or “or” connections within one item [38]. With a pool of 15 items per construct, we went into the item selection stage. Table 1 shows an overview of the five constructs and the sources based on which we created new items for our purpose.

**Table 1:** Constructs, construct definitions, and sources from which items were derived.

Construct	Construct Definition	Initial Items adapted from…
Variability	Degree to which process inputs, steps, or outputs are variable and difficult to predict in advance.	Chang & Tien [55] Whitey et al. [50] Keller [56] Daft & Macintosh [51] Van de Ven & Delbecq [52] + self-created items
Interdependence	Degree to which process participants depend on others in accomplishing their process step.	Chang & Tien [55] Chang & Chiu [53] Gattiker & Goodhue [57] + self-created items
Knowledge- intensity	Degree to which personal judgment as opposed to computational procedures is required during process execution.	Daft & Macintosh [51] Whitey et al. [50] Keller [56] Van de Ven & Delbecq [52] Karimi et al. [58] + self-created items
Differentiation	Degree to which people with different backgrounds, experience, goals, and priorities are involved in executing the process.	Karimi et al. [58] Chang & Chiu [53] Gattiker & Goodhue [57] + self-created items
Importance	Degree to which a process is valuable to the firm and impacting its competitiveness.	Tanriverdi et al. [59] + self-created items

#### Item selection

The aim of the item selection stage was to select items with a high content validity and to drop items that showed only low validity. In this stage, we asked six researchers that had both experience in process management and knowledge about information-processing theory to evaluate the content validity of our items. The experts received a questionnaire in which they were provided with the conceptual definition of each construct and the related item pool. They rated on a five-point Likert Scale, how well each item represents the intended construct. In addition, the experts had the possibility to write comments or questions that helped us to select appropriate items and to further improve them.

For each construct, we selected the six best items based on the highest mean (*M*) and median (*Med*). All items showed a very good content validity (3.6 < *M* < 5; 3.5 < *Med* < 5). The 6 items per construct that have been selected after this stage are displayed in S1 Table.

#### Item revision

In the next stage, we intended to further improve the reliability and validity of the candidate items. We used the index card sorting procedure which has been developed by Moore and Benbasat [40] and that has frequently been applied in instrument development (e.g., see [39, 60]). The card sorting exercise consisted of four rounds in which process management experts were asked to sort the items into categories so that items within one category are very similar and items in different categories are very dissimilar.

As it is recommended to involve subjects that are similar to the target population [39], we included 16 process management experts from the target organization in which we intended to conduct our field study. The experts were process managers, process participants, or process consultants with different levels of process management experiences. We printed all items on cards and started with a sorting example from a different context to make sure that all participants understood the procedure. Then, the experts sorted the items into categories, while we alternated between rounds in which the experts had to identify their own categories (round 1 and 3) and rounds in which the construct categories were provided in advance (round 2 and 4). In the first round, we asked four experts to sort the items into as many categories as they want and to provide a name for each category. In the second round, we asked four different experts to sort the items into the five given construct categories. The third round followed the same procedure as the first round (identifying own categories) while round 4 was equivalent to round 2 (sorting into given categories). In order to show high convergent and discriminant validity, items that belong together should be sorted in the same category while items that measure different constructs should be placed in different categories. All items were improved from one round to the other and items that were continuously misplaced were dropped.

We calculated Fleiss’ kappa [61], a statistical measure of agreement for more than two raters. Table 2 displays the average kappa in all of the card sorting rounds showing that overall, the construct validity increased with the highest kappa reached in round 2 and 4 (where the construct categories have been provided in advance). In the last round, the kappa was higher than 0.61 indicating a substantial agreement [62] and thus, a good inter-rater reliability.

**Table 2:** Average kappa in the different card sorting rounds.

	Round 1	Round 2	Round 3	Round 4
Fleiss Kappa for 4 raters	0.59	0.65	0.61	0.77
Standard error	0.03	0.03	0.04	0.03
95% Confidence interval	0.53 to 0.64	0.578 to 0.71	0.53 to 0.68	0.70 to 0.84

We also asked the experts to indicate if items were difficult to understand and which one they found most easy to judge. Following this procedure, we selected four to five items per construct (see S1 Table).

### Instrument validation

Based on the selected items, we developed a first version of the survey that we pre-tested with ten process management practitioners from the target population. In an interview, we asked the practitioners to read out the survey, to answer the questions, and to share all thoughts or questions they might have. For each interview, we noted all comments and improvement ideas of the participants as well as the time it took them to fill out each section of the questionnaire. This gave us additional information on how the survey could be improved. We revised the wording of the overall questionnaire and kept only four items per construct.

In order to statistically assess the reliability and validity of our instrument, we conducted a pilot test where we invited 43 process practitioners to participate. For the pilot test, we asked participants both from within and outside the target organization based on contacts provided by the central process management team. Participants were asked to evaluate one process they are managing or involved in. Overall, 30 participants replied and filled out the survey. We then conducted an explorative factor analysis to examine the reliability and validity of the overall instrument. We found all of the intended constructs with loadings showing high convergent and discriminant validity. The items that indicated problems in meeting the required validity and reliability thresholds were changed and adapted. The practitioners additionally provided comments which we used to further improve the wording of the questionnaire. The final measurement instrument is provided in S2 Table.

## Instrument application

To demonstrate internal and ecological validity, we applied our measurement instrument in a field survey of process practitioners in a global software development company. Our goal was two-fold: 1) demonstrate the applicability of the measurement instrument by assessing process differences within an organization and 2), identify typical process clusters showing similar patterns of process characteristics.

### Data collection

As we intended to assess differences between organizational processes, the unit of analysis of our study are single work processes. Data was collected through an online survey between January and April 2016 within a large enterprise operating in the software industry. As this was a first test to understand process differences, we intended to avoid getting noisier data by looking at multiple organizations. By looking at one organization a time, we were able to control for other context factors such as organizational or environmental factors, which also influence process management [63]. Instead, we wanted to focus on understanding process variety within one organization, thereby increasing internal validity and providing a tool for internal benchmarking purposes. Software industries usually show a high knowledge-intensity, intense competition, and a high rate of process innovation [64], suggesting increased chances of observing process variety. Moreover, in the software industry, the boundary between the service and the product sector has become fainter and most firms in the software industry straddle both sectors [65]. Thus, the software industry does not only contain a variety of different processes but it is also viewed as a valuable research context in which findings may be of value for companies from other industries, such as high technology firms or manufacturing [64, 66].

### Sample

We asked process practitioners within the company, i.e., process managers, process participants, process consultants, and process stakeholders, i.e., customers and suppliers, to participate in our study and to evaluate one particular process with the help of our questionnaire. To contact potential participants, the target organization provided a list of all nominated process managers, internal process consultants and people with a vested professional interest in process management topics. To motivate participation, we offered insights into the results and sent a reminder two weeks after the initial contact. Overall, we sent an invitation to 451 process practitioners from which 152 filled out our questionnaire (response rate of 33.7%). As our analysis required the absence of missing data, we list-wise deleted cases for which one or multiple process characteristic indicators had missing values. This decreased the sample size for the analysis from 152 to 141, which is less than 10% and should not be a concern as no specific variable was concerned (missing values followed a random pattern) [49].

The process practitioners that participated in our study were from various locations including Africa, America, Asia, and Europe. Most participants were process managers that are responsible for the management of a particular business process (51.97%), the others were process participants (28.29%), consultants (13.16%), and process stakeholders (5.92%). We included process practitioners from different functions of the organization such as strategic business units, customer facing units, human resources, information technology, finance, and product development. Relevant descriptive statistics of our sample population are summarized in Table 3.

**Table 3:** Participant demographics.

Variable	Value	N	Variable	Value	N
**Functional area**	Strategy & Operations Sales	159	**Position**	Process Manager	73
	Services and Support	14		Process Participant	40
	Product Management	21		Process Stakeholder	9
	Finance	30		Process Consultant	18
	Information Technology	5		Missing	1
	Human Resources	32			
	Missing	15			
**Location**	America	21	**Process management experience**	0–1 year	19
	EMEA	94		2–3 years	30
	- Germany	64		4–5 years	22
	- United Kingdom	2		6–7 years	10
	- Czech Republic	2		8–9 years	5
	- Africa	1		10–15 years	25
	- Not specified	25		16–20 years	8
	APJ	2		Missing	22
	Global	4			
	Missing	20			
**Process customer**	Internal	80	**Process type**	Core	52
	External	10		Strategic/Management	21
	Both	51		Support	63
				Unable to judge	5

To make sure that survey participants answered the survey with regards to one dedicated process, the process name and its official identification number had to be stated. By doing so, we made sure that the participants did not answer the survey questions based on their perception of a process but based on a clearly defined process.

### Data analysis

We started by examining validity and reliability of our measurement instrument through a confirmatory factor analysis (CFA) in AMOS. Each item was modeled as a reflective indicator of its hypothesized latent construct and all latent constructs were allowed to co-vary. No identification issues appeared such as very large standard errors for one or more coefficients, negative error variances, or standardized loadings higher than 1 [67]. A solution was found in 9 iterations. The corresponding factor loadings are displayed in Table 4 and the factor correlation matrix (with square roots of AVE on the diagonal) are displayed in Table 5.

**Table 4:** Factor loadings, reliability and average variance extracted.

Construct	Item	Loading	Sig.	Cronbach’s Alpha	Composite Reliability (CR)	Average Variance extracted (AVE)
Variability	PC_Variability_1	0.58	< .001	0.86	0.87	0.63
	PC_Variability_2	0.85	< .001			
	PC_Variability_3	0.84	< .001			
	PC_Variability_4	0.86	< .001			
Interdependence	PC_Interdep_1	0.82	< .001	0.87	0.87	0.62
	PC_Interdep_2	0.84	< .001			
	PC_Interdep_3	0.77	< .001			
	PC_Interdep_4	0.74	< .001			
Knowledge-intensity	PC_Knowledge_1	0.86	< .001	0.91	0.91	0.72
	PC_Knowledge_2	0.76	< .001			
	PC_Knowledge_3	0.87	< .001			
	PC_Knowledge_4	0.89	< .001			
Differentiation	PC_Differentiation_1	0.91	< .001	0.93	0.93	0.76
	PC_Differentiation_2	0.95	< .001			
	PC_Differentiation_3	0.88	< .001			
	PC_Differentiation_4	0.73	< .001			
Importance	PC_Importance_1	0.78	< .001	0.91	0.92	0.73
	PC_Importance_2	0.84	< .001			
	PC_Importance_3	0.93	< .001			
	PC_Importance_4	0.87	< .001			

**Table 5:** Factor correlation matrix (with square roots of AVE on the diagonal).

	Variability	Interdependence	Knowledge -Intensity	Differentiation	Importance
**Variability**	*0*.*79*				
**Interdependence**	0.42	*0*.*79*			
**Knowledge-** **Intensity**	0.54	0.36	*0*.*85*		
**Differentiation**	0.31	0.53	0.25	*0*.*87*	
**Importance**	0.16	0.34	0.21	0.42	*0*.*85*

According to Hair et al. [49], all indicator loadings should be at least .5, and ideally .7 or higher. All our measures met these thresholds and were significant at p < .001, with only PC_Variability_1 showing a relatively low loading. Overall, the measures seem to be strongly related to their associated construct. Likewise, all constructs showed sufficient reliability (Cronbach`s > 0.7 and CR > 0.5) [68].

Convergent validity was tested using three criteria of Fornell and Larcker [69]. First, as can be seen from Table 4, all item loadings were significant and all but one exceeded the threshold of 0.6. Second, construct composite reliability exceeded 0.8. Third, average variance extracted (AVE) was higher than 0.5. Thus, also convergent validity was ensured.

Discriminant validity was tested analyzing the AVE for each construct as compared to the squared correlation between that and any other construct. As can be seen from Table 5, the smallest AVE (AVEInterdependence = 0.63) was greater than the largest squared correlation (r^2^ Variability, Knowledge-Intensity = 0.29).

We also checked for common method bias [70] by conducting an explorative factor analysis including all indicators in SPSS. The first factor extracted 35% of the variance, which suggests that common method bias may not be substantial. In addition, we included a latent common method factor in AMOS to examine if the fit increased significantly through adding the new factor. This was, however, not the case which is why we conclude that common method bias is not a substantial issue in our study.

Overall, the analysis demonstrated reliability and validity of our developed measurement instrument. Goodness of fit statistics for the overall structural model (GFI = 0.84, CFI = 0.93, RMSEA = 0.08, χ2 = 293, df = 160, p = 0.00) suggest reasonable fit of the model to the data [49]. Some residual error existed in the model that resulted from item PC_Variability_1 showing a relatively low loading (.58) which was, however, still significant (p < .001) and reached the threshold of .5 [49]. Excluding the item did not improve our model significantly and the overall reliability and validity remained good, so we kept the item in the measurement model.

## Post-hoc analysis

To examine our measurement results in more detail through post-hoc tests, we conducted a cluster analysis which can be used to test for similarities and dissimilarities in the data objects [71]. We combined hierarchical and non-hierarchical methods as recommended by Hair et al. [49] and Punj and Stewart [72]: we used hierarchical cluster analysis to establish the number of clusters and initial cluster centroids, and then used these as input for the non-hierarchical cluster analysis. Similar to the approach of Bensaou and Venkatraman [73] and Premkumar, Ramamurthy, and Saunders [74], we used 1) standardized values for each variable, 2) the Ward algorithm and 3) the squared Euclidean distance measure for the cluster analysis.

To determine the number of clusters, we used the Agglomeration Coefficient [49]. This coefficient provides information about the similarity and dissimilarity of two clusters that are combined at each stage of the cluster analysis. A small coefficient means that similar clusters or cases are combined. A large increase in the coefficient suggests that dissimilar clusters have been combined which represents a good cutoff point [49]. Usually, the two-cluster solution has a very high change in agglomeration coefficient but provides less interesting findings, which is why it is recommended to examine the next highest agglomeration coefficient [49]. Table 6 shows the percentage changes in the agglomeration coefficient from one stage to the other.

**Table 6:** Agglomeration coefficients and coefficient change for different cluster solutions.

Stage	Cluster1	Cluster2	Coefficient	Number of Clusters	Difference	Proportional increase in heterogeneity to next stage (in %)
…	…	…	…	…	…	…
131	2	7	209.38	10	13.32	0.06
132	16	26	222.70	9	14.31	0.06
133	1	25	237.01	8	20.73	0.09
134	2	4	257.74	7	20.94	0.08
135	28	46	278.68	6	33.38	0.12
136	28	56	312.06	5	38.69	0.12
137	16	84	350.75	4	47.99	0.14
138	1	16	398.74	3	64.87	0.16
139	1	2	463.61	2	179.72	0.39
140	1	28	643.33	1	-	-

**Fig 2:**
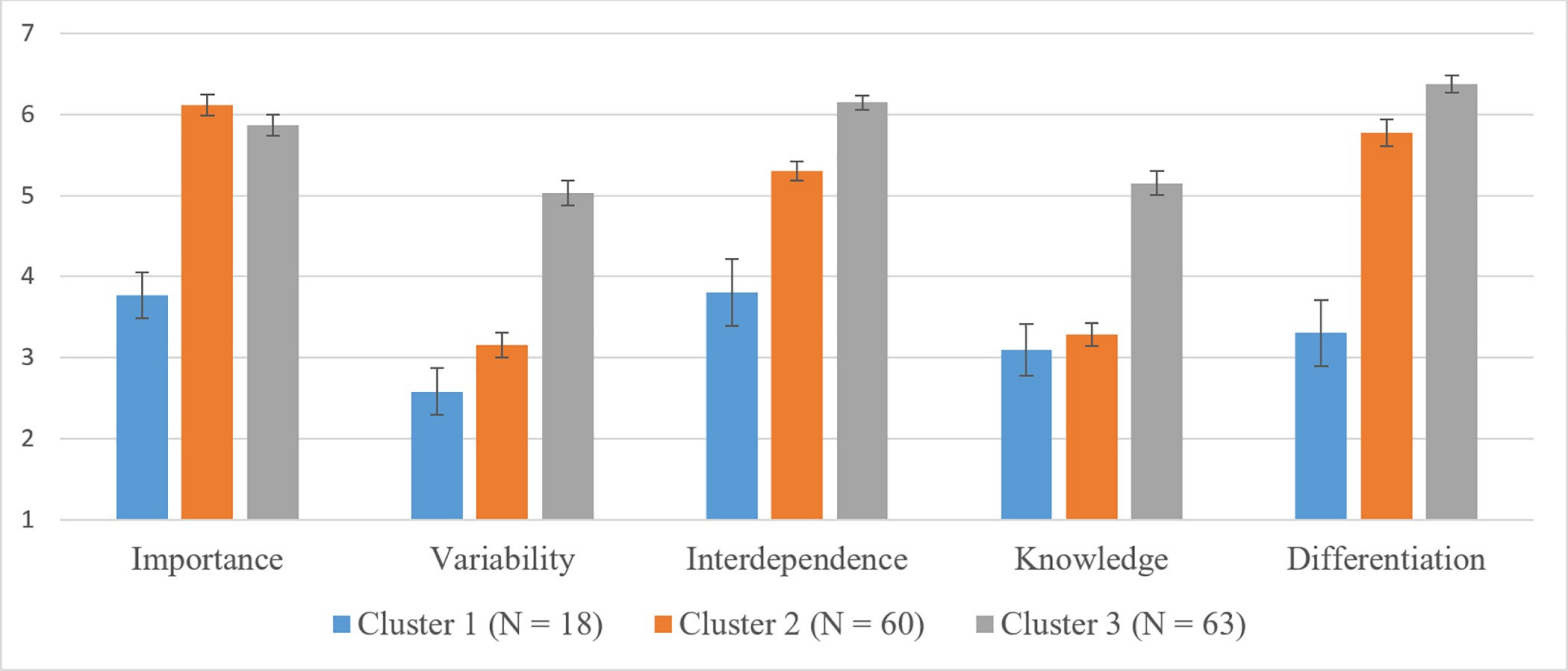
Results of cluster analysis. Cluster means and standard errors.

As the change from a three to a two-cluster solution is relatively high, we decided to derive three process clusters. Building on recommended procedures [49, 72], we saved the cluster means of the hierarchical analysis and used them as initial seed for the K-Means cluster analysis. The final results (cluster means, mean square, and F-value) are displayed in Table 7 and shown in a graphical way in Fig 2.

**Table 7:** Cluster means and results of ANOVA.

	Cluster Means				
	Cluster 1	Cluster 2	Cluster 3	Mean Square	F (sig)
Importance	3,76	6,12	5,87	39.88	37.58 (< .001)
Variability	2,58	3,15	5,03	72.33	48.72 (< .001)
Interdependence	3,81	5,31	6,15	40.28	42.37 (< .001)
Knowledge	3,10	3,28	5,15	64.10	46.37 (< .001)
Differentiation	3,31	5,77	6,27	65.97	47.43 (< .001)

Our post-hoc analysis suggests the existence of three clusters: Cluster 1 contains processes that have low values on all process dimensions. In our data, these were rather administrative, structured processes such as *annual performance review*, *annual compensation review*, or *manage travel and expenses*. Based on an information-processing perspective, these processes are likely to have low information processing requirements. We call them “processes with low information-processing requirements” due to their high analyzability and low levels of dependencies.

Second, we identified one cluster with a mixed picture. This cluster showed low levels of variability and knowledge-intensity but high levels of importance, interdependence and differentiation. Example processes of this process cluster are *recruiting*, *internal audit*, *and market intelligence & benchmarking*. According to information processing-theory, these processes should have medium levels of information processing requirements. We refer to them as “processes with medium information-processing requirements” due to their high dependency on other teams but their relatively high analyzability (low knowledge-intensity).

Last, cluster 3 includes processes that had high values on all process dimensions. These are the processes which, according to theory, are of high information processing requirements. Example processes of our sample are the more strategic, knowledge-intensive processes such as *deal approval*, *deal support*, *innovation lifecycle*, *or post-merger integration*. Due to the high amount of knowledge and variability of these processes as well as the high interdependencies to various different teams, we call them “processes with high information-processing requirements”.

### Cluster evaluation

We evaluated the cluster analysis in three different ways: First, we checked the stability of the cluster solution as the results of cluster analysis are highly dependent on the approach that is taken by the researcher [49]. Second, we examined to what extent our proposed process clusters relate to varying information-processing requirements which we would hypothesize based on information-processing theory. Third, we compared the clusters to the most frequently applied process differentiation into core, management, and support processes [3] to understand how far both frameworks can be used to analyze and explain process management.

#### Stability

As the results obtained through cluster analysis can vary depending on many factors such as the centroids used as an input or the order of the data points, we validated our cluster solution with additional cluster analysis of the same sample [49]. We performed one cluster analysis without providing the cluster centroids of the hierarchical analysis as an input and one where we sorted the dataset randomly in another way. In both cases, we identified the same cluster solution with almost identical number of data points in each cluster. Cross-tabs showed that the cases that were clustered together in our first approach were still clustered together in the second, and third cluster analysis which supports cluster stability.

#### Information-processing requirements

As our process classification model is based on information-processing theory, the resulting process clusters should differ in their information-processing requirements which consist of the *amount* of information processing that is required to execute the process as well as the degree to which information is ambiguous and needs to be *interpreted* [36]. As a control variable, we included two items measuring information-processing requirements (“*The process requires a significant amount of information processing”* and “*The information used in making decisions during process execution can be interpreted in different ways*”), both measured on a seven-point Likert Scale from 1 (“fully disagree”) to 7 (“fully agree”). A one-way ANOVA showed that the three process clusters differ in the amount of information processing (*F*(2,138) = 4.84, p = .009) and the degree of information interpretation (*F*(2,138) = 11.45, p < .001). Planned contrasts showed that cluster 1 requires less information processing than cluster 2 and 3 (t(138) = 2.87, p = .005; t(138) = 2.98, p = .003). Between cluster 2 and 3, there is no difference in the amount of information processing (t(138) = 0.14, p = .887). In addition, cluster 3 showed a significant higher need for information interpretation than cluster 1 and cluster 2 (t(138) = 3.43, p = .001; t(138) = 4.28, p < .001). Between cluster 1 and 2, no difference could be observed (t(138) = 0.54, p = .591). Thus, in alignment with information-processing theory, we observed differences in information-processing requirements between the three process clusters.

#### Comparison to existing process frameworks

One purpose of systematically developing a process classification model based on a) process dimensions and b) theoretical perspectives was to explain distinct process management requirements. Compared to existing process frameworks developed in practice, we argue that an understanding of underlying process dimensions is crucial to derive management recommendations for different types of processes. Thus, we analyzed to what extent our process clusters, which are based on process dimensions, are managed in different ways.

To do so, we needed to find an appropriate operationalization of “process management”. As there is a plethora of different management dimensions, we selected a wide variety from different sources [12, 36, 44, 57, 75, 76]. We settled on six:

**Hierarchy** describes management approaches in which problems are referred to higher level managers to make a decision. In such a centralized approach, higher level management is involved in employees tasks while, in a decentralized approach, employees have control over their tasks [75].

**Goal setting** describes the extent to which goals are set to guide employees actions [12, 36]. In a process management context, goal setting describes the extent to which goals are set to steer process participant’s behavior.

The implementation of **rules** is one basic practice which can be formalized in official process documentation. This dimension is often described as formalization in general management theory [75, 76]. It describes the degree to which written rules, procedures, and regulations are provided to employees [75].

**Self-containment** describes the formation of groups of people that are responsible for one or few outputs and are free to decide upon the exact execution [12, 36]. This management dimension describes the degree to which the process (or parts of the process) can be performed autonomously.

**Information systems (IS) investments** show the extent to which process execution is supported through information systems. Investments in information systems can help to collect and distribute information. However, it has been shown that these investments are not supportive in all situations [44, 57].

**Lateral relations** are relationships that are being built to connect people or departments within the organization. Typical forms of lateral relations include liaison roles or integrator roles that foster collaboration across lines of authority [12, 36].

Measurement items for these constructs were developed in the same way as the process dimensions, with rounds of ranking exercises, card sorting, pre-test, and pilot-test (see Fig 1). Items for these constructs are displayed in the S3 Table.

We conducted ANOVAs with the process management constructs as the dependent variable and the process cluster as the independent variable and found that the process clusters differed in 4 out of 6 management dimensions as displayed in Table 8. We compared the results to two other types of process taxonomies: the frequently used differentiation into core, management, and support processes [3] as well as more functional differentiations similar to the framework of APQC [28]. For this, survey participants assessed the general nature of the process [1) core process of the organization (directly contributing to the organizational value creation), 2) strategic management process of the organization (focusing on strategic tasks, monitoring, and control of other processes, 3) support processes of the organization (enabling and supporting the core processes of the organization)] as well as which functional area the process belonged to (Strategy & Operations, Sales, Services and Support, Product Management, Finance, Information Technology, or Human Resources) (see S4 Table).

**Table 8:** Differences in the management approach between different process classification models.

			Core, management, and support process classification				Functional process classification (HR, IT, Finance, etc.)				Our classification model (Cluster I, II, III)			
		df	Mean Square	F	p	df		Mean Square	F	p	df	Mean Square	F	p
Hierarchy	BS	2	1.16	0.46	.632	6		3.33	1.29	.266	2	9.70	4.03	**.020**
	WS	124	2.52			119		2.58			129	2.40		
Goal setting	BS	2	1.59	0.78	.461	6		4.52	2.40	**.032**	2	7.72	4.00	**.021**
	WS	123	2.04			118		1.88			128	1.93		
Rules	BS	2	1.63	0.61	.545	6		4.61	1.83	.100	2	8.00	3.21	**.044**
	WS	124	2.66			119		2.53			129	2.49		
Self-containment	BS	2	7.45	3.04	.052	6		3.29	1.35	.242	2	1.14	0.45	.636
	WS	124	2.45			119		2.45			129	2.51		
IS investments	BS	2	2.76	0.96	.386	6		3.21	1.09	.375	2	3.32	1.16	.317
	WS	123	2.88			118		2.96			128	2.86		
Lateral relations	BS	2	1.33	0.84	.434	6		0.87	0.54	.780	2	5.04	3.17	**.045**
	WS	124	1.58			119		1.63						

The differentiation into core, management, and support processes did not reveal any differences in management between the processes. The functional process classification showed management differences in one of the six management dimensions, while our classification model showed management differences in four out of six dimensions.

**Fig 3:**
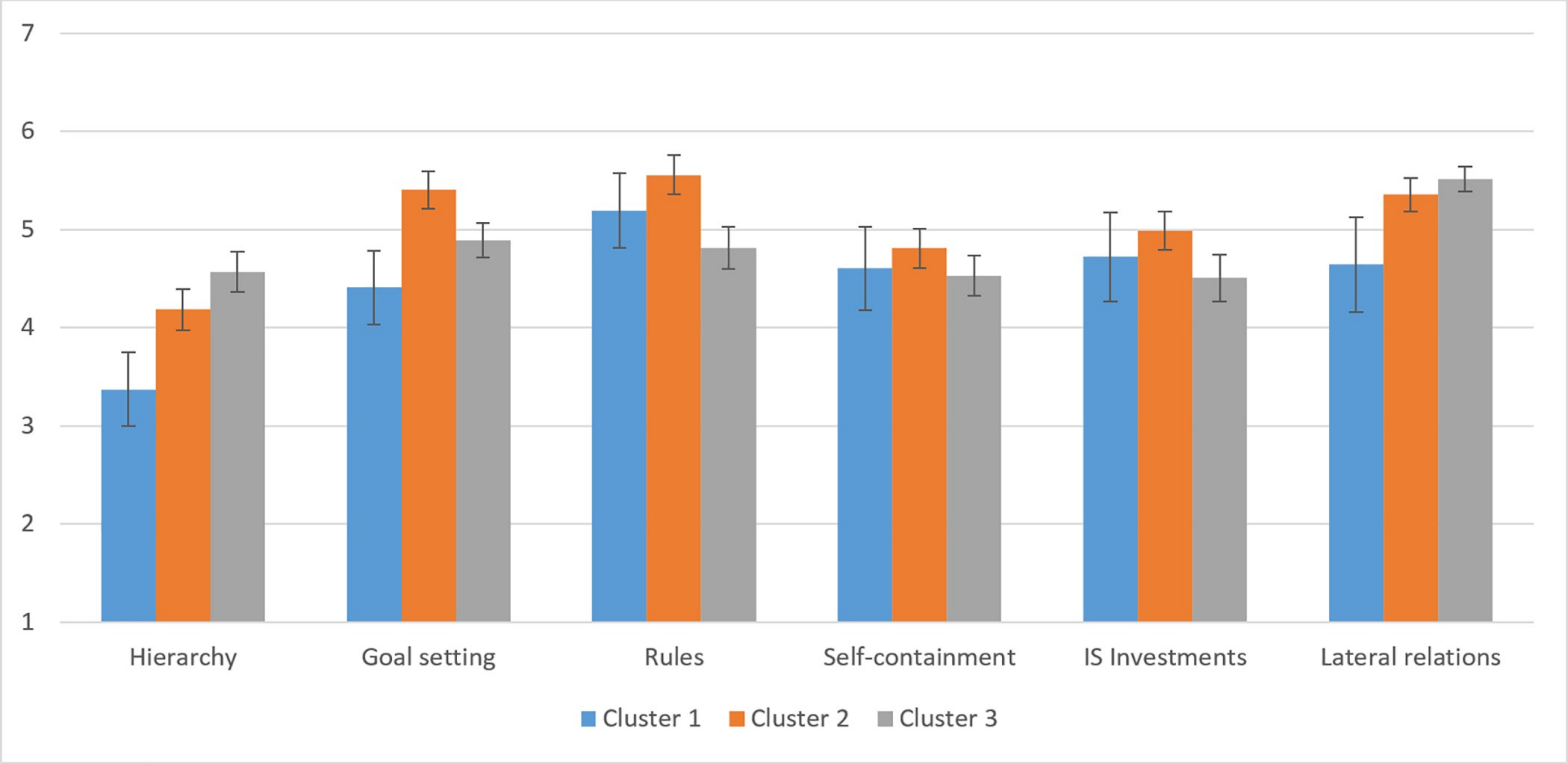
Management differences between cluster 1, 2 and 3 processes.

In Fig 3, the direction of the effects of process clusters on management is displayed. With an increase in information processing requirements (Cluster 1 < Cluster 2 < Cluster 3), the degree of centralization in terms of a hierarchy increases. Setting goals to steer process execution seems to be particularly important for processes belonging to cluster 2. The same is true for setting rules and standard procedures. Process managers of cluster 3 processes apply less rules and procedures. Last, lateral relations become particularly important for processes with higher information processing requirements, such as processes of cluster 2 and 3. For the degree of self-contained tasks or the investments in information systems, no effects have been identified.

The functional process differentiation revealed only differences in the goal setting dimension, which was caused by the difference between Sales and Human Resources processes: In Human Resources processes, goals were set to a greater extent to guide process execution (t(38) = 2.54, p = .015).

## Discussion

In this paper, we developed a new *process classification model* and *an instrument* to measure process variety, which we believe may better support decision-making and the identification of appropriate management recommendations. We differentiated processes based on five underlying process dimensions and operationalized each of these dimensions in a reliable and valid survey instrument (S2 Table):

- **Variability** describes the degree to which process inputs, steps, or outputs are variable and difficult to predict in advance.
- **Interdependence** describes the degree to which process participants depend on others in accomplishing their process step.
- **Knowledge-intensity** describes the degree to which personal judgment as opposed to computational procedures is required during process execution.
- **Differentiation** describes the degree to which people with different backgrounds, experience, goals, and priorities are involved in executing the process.
- **Importance** describes the degree to which a process is valuable to the firm and impacting its competitiveness.

We also demonstrated that these dimensions can be used to identify typical cluster of processes, and that such a categorization is more useful for explaining distinct management approaches as compared to existing process frameworks. Overall, we identified three different types of processes:

- **Cluster 1**: Processes with low information-processing requirements showing low levels on all process dimensions (these processes require less information processing and less information interpretation),
- **Cluster 2**: Processes with medium information-processing requirements showing low levels on variability and knowledge-intensity, but high levels on interdependence, importance, and differentiation (these processes require more information processing but not much information interpretation), and
- **Cluster 3**: Processes with high information-processing requirements showing high levels on all process dimensions (these processes require both more information processing and more information interpretation).

As our examination of information-processing requirements revealed, cluster 2 and 3 processes required an increased amount of information compared to cluster 1. In addition, cluster 3 processes required and increased amount of information interpretation as compared to cluster 1 and 2. Thus, with an increase in process interdependence, differentiation, and importance the need for information increased, while with an increase in knowledge-intensity and variability, the need for information interpretation increased. When there are a lot of different process participants involved, which highly depend on each other in executing an important process (as for cluster 2 processes), the amount of information that process participants need to process increases. So, process participants establish rules to be followed. If, in addition, process participants encounter a lot of different situations and they need to apply personal judgment (as for cluster 3 processes), the interpretation of information increases. So, process participants cannot use standardized rules but they rather rely on higher level management for coordination.

Theoretically, processes with medium levels of information-processing requirements could also result based on a different pattern of process characteristics, e.g. processes with low levels of interdependence, importance and differentiation but high levels of variability and knowledge-intensity. Checking the raw data, we indeed identified such cases like managing the internal social media platform or providing contract management services. However, as there were only few such cases, they have not been detected by our cluster analysis (see [49]). Therefore, future research might identify more process clusters also showing medium levels of information-processing requirements but a different pattern of process characteristics.

### Implications for research

In this paper, we contribute to research in the field of process management by introducing a new understanding of process variety which is a) based on underlying process dimensions, b) linked to theory, and c) systematically derived based on empirical findings. This understanding of process variety is a valid alternative for the many process frameworks developed in practice or the attempts to conceptualize process differences in process management theory which lack theoretical and/or empirical basis. Thus, our paper deepens the understanding of organizational process differences.

A conceptualization of process differences can guide future research in the field of process management allowing researchers to better describe and compare organizational processes, to explain contradictory findings in process management research, and to identify additional process types. For example, the suggested process classification model enables future research to more specifically describe the processes in scope of a research study by applying our measurement instrument or process categorization. All process dimensions have been systematically derived from OIPT and operationalized for the field of process management. Describing processes on the various dimensions increases the insights gained from a study as well as the comparability of research findings. In addition, the nature of a process can now be operationalized through the survey instrument developed in this paper and can then be considered as an additional factor in research studies. The wording of several of our measurement items setting (e.g. “process steps vary a lot”) were chosen to be relative rather than in absolute terms such that they can fit various settings and can be applied to a variety of different organizations also helping to compare processes within one organization (internal benchmarking). Process differences can be included as moderating factors, contributing to a contingency approach in process management which explains why certain process management practices have proven successful for some processes but not for others. This helps to understand when (for which processes) certain process management practices are successful or not [77].

Our findings also provide new explanations for existing research findings, e.g. why process complexity negatively influences process standardization [4]. Processes that are considered as complex are typically processes that would be classified as processes with high information-processing requirements based on our classification model. According to OIPT and based on our findings, these processes show high levels of information-processing requirements, so they require management methods that enhance and support information-processing. Rules and defined procedures, as is the case of process standardization initiatives, are not capable to deal with the increased information-processing requirements [12] providing a new explanation for existing research findings. The application of OIPT to the level of organizational processes demonstrates a new application field for the theory, thereby contributing to research on OIPT.

Last, research can build on our findings and evaluate or enhance our process classification model. As an example, future research can apply our instrument to measure process differences in further contexts (e.g., in other industries, with other data sources, etc.). By doing so, the external validity of our instrument can be evaluated. It can be examined, for example, whether the process types are only valid in certain industries or differ between industries. In addition, it can be examined if additional process types exist in other contexts.

### Implications for decision-making

Our findings also have implications for process management practice in supporting organizational decision-making. In this paper, we demonstrate that there is a variety of processes with distinct process management requirements: Some processes are highly interdependent, involve various different organizational departments, are highly variable, require much knowledge during process execution, and are very important to realize strategic goals. These processes have different process management requirements than processes that don´t involve much knowledge, that are rather repetitive, and that can be performed without depending on many other departments. While the plethora of existing process frameworks developed in practice cannot address this diversity of processes, the process classification model developed in this paper takes these differences into consideration and provides a strategic instrument for both organizational decision-makers and individual process managers.

From an organizational perspective, the proposed process classification model can serve as a strategic instrument to distinguish processes in organizations, to prioritize initiatives, and to plan projects. As such, it provides an alternative to the various process frameworks applied in practice, like the differentiation into core, management, and support processes [3] or functional process descriptions [28]. When creating an overview of organizational processes, process management experts can use our classification model as a basis to derive strategic goals, prioritize initiatives, or plan improvement projects. The classification of processes could either be done via a rigorous survey methodology, in a workshop, or by each individual process manager for the process she is responsible for. In addition, the type of process can be one aspect in selecting appropriate process maturity models [78] or new maturity models can be developed that suggest certain maturity criteria as more or less important, depending on the type of process that has to be managed.

In addition, the proposed process classification model can support individual process managers in making decisions with respect to the management of single processes. Every process has different process management requirements and a process manager responsible for one particular process needs to identify appropriate management practices. We identified, for example, that processes which are highly interdependent, involve various different organizational departments, are highly variable, require much knowledge during process execution, and are very important to realize strategic goals (cluster 3 processes), require less formal rules, often have management involved in decision-making and are supported by lateral relations which help to coordinate between process participants. These management requirements differ for processes that don´t involve much knowledge, that are rather repetitive, and that can be performed without depending on many other departments. Building on our process classification model, process managers can develop additional management recommendations for each type of process. The derived process cluster differ in their information-processing requirements and need management methods that provide information and/or support the interpretation of information. Thus, process management experts can examine to what extent process management practices address information-processing requirements and so, which process management practices are appropriate for which process type.

Overall, identifying a distinct management approach for organizational processes based on our process classification model has the potential to increase the efficiency and effectiveness of process management in organizations.

### Limitations

Our study is not without limitations. First, we examined our measurement instrument in only one case organization. While we chose to involve one case organization to be able to focus on process differences within one organization and to control for other factors that potentially influence process management [63], we suggest that future research uses our instrument and applies it in other organizations to demonstrate external validity. As we involved a broad variety of organizational departments which also exist in other organizations, we believe that an application in other organizations is possible and likely to provide similar insights. In addition, researchers have discussed that software industries share many characteristics with both the product and the service sector, making our findings potentially relevant also for other industries [64, 66].

Second, while reliability and validity of the measurement instrument have been demonstrated, some goodness of fit statistics, in particular the GFI (0.84) and the significant χ2 test, may suggest that potential alternative re-specifications of the model could further improve fit to the data [79].

Third, as stated under implications for research, we worded several items in our measurement instrument in relative rather than absolute terms, avoiding objective benchmarks (e.g. “process steps vary a lot”, "participants are highly dependent on other participants”). This allows for wider applicability of the metrics yet also induces potential response bias as the individual response anchor may vary. In other words, the evaluation of process characteristics may be influenced by the perception of process experts who might compare the process to other processes in the organization. While this approach is important for internal benchmarking and prioritization of resources within one organization, future research should also look into alternative, more objective ways of measuring process characteristics, which might be particularly important for cross-organizational research.

Fourth, we provided only first insights that the understanding of process variety developed in this paper can be more helpful in differentiating between process management practices compared to existing process frameworks developed in process management practice. However, there are many aspects of process management and future research should analyze additional management practices and link them to process differences. In addition, it has to be examined to what extent our understanding of process variety should replace existing frameworks or if they rather should be combined. While the differentiation into core, management, and support processes makes sense to structure an organization on a high level, our classification model makes sense when it comes to deriving appropriate management recommendations for processes on a lower level. It might be that also combinations of process taxonomies provide value for organizations. This is to be examined in future research.

Fifth, we only consider process differences in this paper and argue that an understanding of the dimensions of processes is crucial for decision-making in process management. However, research has also discussed organizational factors, environmental factors, or process management goals which influence process management [63]. Thus, decision-makers should not only understand process differences but also differences in organizations, environments, and goals. A holistic management approach would require to also examine other factors and integrate them into more comprehensive decision support systems. Related to this, it might be of value to establish some kind of nomological network for process differences, examining what causes process differences and how do process differences related to other organizational or environmental variables.

## Conclusion

Understanding process differences based on underlying process dimensions is a valuable approach for moving towards a contingency perspective in the process management literature. As processes in organizations are highly variable, we first need to understand why (i.e., on which dimensions) they are different to then derive appropriate management recommendations. The process dimensions we suggest in this paper build on information-processing theory and contribute to a more differentiated view on processes and process management practices. As demonstrated in this paper, our understanding of process variety and the developed measurement instrument of process variety provides a valuable alternative to the plethora of process frameworks that have been developed in process management practice but that have failed to support decision-making in process management.

## Supporting information

S1 Table Stepwise item adaptation. (PDF)

S2 Table Final items to measure process variety. (PDF)

S3 Table Final items to measure process management practices. (PDF)

S4 Table Control variables. (PDF)
