# Supplementary material for: Development and validation of an instrument to measure and manage organizational process variety
Source: PLoS One. 2018 Oct 23;13(10):e0206198. doi: 10.1371/journal.pone.0206198 (PMC6198977; doi:10.1371/journal.pone.0206198)
Supplement: S1 Table — (PDF) [file pone.0206198.s001.pdf]

S1 Table. Stepwise item adaptation.

Items selected after ranking exercise	**Item revisions after index card sorting exercise** (items selected for pre-test are marked with*)
**Variability:**	
There are frequent exceptions expected to arise during process execution.	There are frequent exceptions expected to arise during process execution.
It is difficult to predict the output of the process.	It is difficult to predict the steps and output of the process.*
The process is highly variable.	The process is highly variable.*
Process participants mainly follow the same process steps from one time to the other (-).	Process participants need to follow different process steps from one instance of the process to another.*
Process execution can vary a lot from one time to the other.	Process execution can vary a lot from one instance of the process to another.*
Process participants need to be flexible and adapt to the process conditions.	There are a lot of different situations or problems expected to encounter during process execution.
**Interdependence:**	
Process participants highly depend on others to obtain resources or information.	Process participants highly depend on others to obtain resources or information.*
Process participants are highly dependent on others in executing the process.	Process participants are highly dependent on others in executing the process.*
Frequent information exchanges between process participants are essential for the process execution.	Frequent information exchanges between process participants happen during process execution.*
Close coordination between process participants is essential for the process execution.	Close coordination between process participants is essential for the process execution.*
The interdependence of process participants is high.	The interdependence of process participants is high.
The number of interactions between process participants is high.	The number of interfaces between process participants is high.
**Knowledge-intensity:**	
The process execution requires a lot of knowledge.	The process execution requires a lot of knowledge. *
Executing the process requires a lot of personal experience.	Process participants need to rely more on personal experience than on standard procedures.*
Personal judgment highly influences process outcomes.	Personal judgment highly influences process outcomes.
The process execution requires creative thinking of the process participants.	Process participants cannot apply computational procedures to accomplish their process steps.*
The process execution requires a lot of personal judgment.	The process execution requires a lot of personal judgment.*
The process is influenced by personal decision-making of process participants.	Decision-making of process participants is influencing process execution.
**Differentiation:**	
The process cuts across multiple functional units.	There are very different functional units involved during process execution.
All process participants have very different backgrounds.	All process participants have very different educational backgrounds.*
The process involves process participants with different.	The process involves process participants with very different functional backgrounds.*
The experience of the people involved in the process differs a lot.	The experience of process participants differs a lot.
Process participants are from different areas of expertise.	Process participants are from very different areas of expertise.*
Process participants have a variety of different experiences.	Process participants have a variety of different experiences.*
**Importance:**	
The process is valuable to our firm.	The process is very valuable to our company.*
The process is unique to our firm.	The process is very unique to our company.
The process is common among our competitors (-).	The process is common among our competitors.*
In our industry, there are equally effective alternative ways of doing the process.	The process is very important to our company.*
It is very difficult for our competitors to replicate how we do the process.	It is very difficult for our competitors to replicate how we do the process.*
It would be very costly for our competitors to imitate the process.	It would be very costly for our competitors to imitate the process.
