# Supplementary material for: Development and validation of an instrument to measure and manage organizational process variety
Source: PLoS One. 2018 Oct 23;13(10):e0206198. doi: 10.1371/journal.pone.0206198 (PMC6198977; doi:10.1371/journal.pone.0206198)
Supplement: S2 Table — (PDF) [file pone.0206198.s002.pdf]

S2 Table. Final items to measure process variety.

**Construct**	**Item Code**	**Items** (1 = fully disagree, …, 7 = fully agree)
Variability	PC_Variability_1	Process participants need to follow different process steps from one instance of the process to another.
	PC_Variability_2	Process steps are difficult to predict as they can change from one instance of the process to another.
	PC_Variability_3	Process execution can vary from one instance of the process to another.
	PC_Variability_4	Process steps vary a lot from case to case.
Interdependence	PC_Interdep_1	Process participants are highly dependent on other participants for process execution.
	PC_Interdep_2	Process execution requires very close coordination between process participants.
	PC_Interdep_3	The interdependencies between the steps in the process are high.
	PC_Interdep_4	Process participants highly depend on other participants to get required resources.
Knowledge-intensity	PC_Knowledge_1	Process participants need to rely more on experience than on standard procedures.
	PC_Knowledge_2	Process execution requires knowledge rather than following standard procedures.
	PC_Knowledge_3	Instead of applying standard procedures, process participants need to use their personal judgment.
	PC_Knowledge_4	Process execution requires participants to make decisions based on experience rather than based on standard procedures.
Differentiation	PC_Differentiation_1	The process involves process participants from different functions.
	PC_Differentiation_2	Process participants are from different areas of expertise.
	PC_Differentiation_3	Process participants have different functional backgrounds.
	PC_Differentiation_4	Process participants have a variety of different job experiences.
Importance	PC_Importance_1	The process is highly valuable to our company.
	PC_Importance_2	The process is of high strategic importance to our company.
	PC_Importance_3	The process contributes significantly to the value creation of our company.
	PC_Importance_4	The process contributes significantly to the competitiveness of our company.
