# Supplementary material for: Development and validation of an instrument to measure and manage organizational process variety
Source: PLoS One. 2018 Oct 23;13(10):e0206198. doi: 10.1371/journal.pone.0206198 (PMC6198977; doi:10.1371/journal.pone.0206198)
Supplement: S3 Table — (PDF) [file pone.0206198.s003.pdf]

S3 Table. Final items to measure process management practices.

**Construct**	**Item Code**	**Items** (1 = fully disagree, …, 7 = fully agree)
Hierarchy	Hierarchy_1	Decisions about a process problem cannot be solved without managers.
	Hierarchy_2	Problems occurring within the process are taken to higher level managers to reach a decision.
	Hierarchy_3	Process participants reach out to higher level managers to make decisions about a process problem.
Goal Setting	Goals_1	Goals for process participants are set to ensure that process execution goes into the right direction.
	Goals_2	Goals for process participants are set to guide process execution.
	Goals_3	Setting goals for process participants ensures that the desired outcomes are achieved during process execution.
Rules	Rules_1	The relevant process steps are defined in rules and procedures.
	Rules_2	Details of the process execution are summarized in rules or defined procedures.
	Rules_3	Process execution is driven by rules and defined procedures.
Selfcontainment	selfcontained_1	We arranged all tasks within the process, so that process participants can work self-contained (i.e. they have all necessary resources and do not depend on others).
	selfcontained_2	We structured the process in a way that units of work can be performed autonomously.
	selfcontained_3	We ensured that process participants can work autonomously.
IS Investments	Isinvest_1	Our investments in information systems help to streamline process activities.
	Isinvest_2	We have committed considerable time and money for information systems that support the process.
	Isinvest_3	In the past 2 years, we have upgraded the information systems that support the process.
Lateral Relations	Lateralrelation_1	When problems occur, process participants solve them through directly reaching out to colleagues in other functional units.
	Lateralrelation_2	Process participants directly contact the colleagues in other units when they need information.
	Lateralrelation_3	If required, process participants from different functional units contact each other directly during process execution.
