# Supplementary material for: Development and validation of an instrument to measure and manage organizational process variety
Source: PLoS One. 2018 Oct 23;13(10):e0206198. doi: 10.1371/journal.pone.0206198 (PMC6198977; doi:10.1371/journal.pone.0206198)
Supplement: S4 Table — (PDF) [file pone.0206198.s004.pdf]

S4 Table. Control variables.

Construct	Item Code	Items
Respondent's Position	Control_Position	What is your position within the process?<br>(1 - Process Manager, 2 - Participant, 3 - Stakeholder, 4 - Others)
Process Nature	Control_Nature	What is the general nature of this process?<br>(1 - Core process, 2 - Strategic management process, 3 - Support process, 4 - I don`t know / I cannot judge)
Process Customers	Control_Customers	What kind of customers does the process serve?<br>(1 - Internal, 2 - External, 3 - Both)
Process Frequency	Control_Frequency	How often does the process run in your company?<br>(6 - Several times per hour, 5 - Several times per day, 4 - Several times per week, 3 - Several times per month, 2 - Several times per year, 1 - Once per year, 0 – Less frequent)
Process Duration	Control_Duration	How long does it usually take to run through the process?<br>(1 - Less than several minutes, 2 - Several minutes, 3 - Several hours, 4 - Several days, 5 - Several weeks, 6 - Several months, 7 - A whole year, 8 - More than a year)
Respondent's Experience in BPM	Control_Experience in BPM	How long have you been working in a process management role over your working life?<br>(in years)
Respondent's Functional Area	Control_FunctionalArea	Which Functional Area do you work for?<br>(Strategy & Operations, Sales, Services and Support, Product Management, Finance, Information Technology, or Human Resources)
Respondent's Location	Control_Location	In which geographical location are you working?<br>(text)
Information-Processing Requirements	Control_IPR_1	The process requires a significant amount of information processing.<br>(1 = fully disagree, …, 7 = fully agree)
	Control_IPR_2	The information used in making decisions during process execution can be interpreted in different ways.<br>(1 = fully disagree, …, 7 = fully agree)
